# Computer Based Melanocytic and Nevus Image Enhancement and Segmentation

**DOI:** 10.1155/2016/2082589

**Published:** 2016-09-28

**Authors:** Uzma Jamil, M. Usman Akram, Shehzad Khalid, Sarmad Abbas, Kashif Saleem

**Affiliations:** ^1^Department of Computer Engineering, Bahria University, Islamabad, Pakistan; ^2^Government College University, Faisalabad, Pakistan; ^3^National University of Sciences & Technology, Islamabad, Pakistan

## Abstract

Digital dermoscopy aids dermatologists in monitoring potentially cancerous skin lesions. Melanoma is the 5th common form of skin cancer that is rare but the most dangerous. Melanoma is curable if it is detected at an early stage. Automated segmentation of cancerous lesion from normal skin is the most critical yet tricky part in computerized lesion detection and classification. The effectiveness and accuracy of lesion classification are critically dependent on the quality of lesion segmentation. In this paper, we have proposed a novel approach that can automatically preprocess the image and then segment the lesion. The system filters unwanted artifacts including hairs, gel, bubbles, and specular reflection. A novel approach is presented using the concept of wavelets for detection and inpainting the hairs present in the cancer images. The contrast of lesion with the skin is enhanced using adaptive sigmoidal function that takes care of the localized intensity distribution within a given lesion's images. We then present a segmentation approach to precisely segment the lesion from the background. The proposed approach is tested on the European database of dermoscopic images. Results are compared with the competitors to demonstrate the superiority of the suggested approach.

## 1. Introduction

Skin cancer early detection is very important because of its successful and economic treatment. Skin cancer is the most common form of cancer which is rapidly increasing in recent decades [1]. Malignant melanoma (MM) is the most common type of skin cancer which is usually found in white skin people but has also been rarely seen in the dark skin individuals. Early stage diagnosis of skin cancer is extremely critical for its treatment. Amongst various types of skin cancer, melanoma is one of the most fatal diseases which has the highest death rate. Melanoma originates in melanocytes which are the cells in the skin that produce pigment or melanin. It mostly occurs on those parts of the human body that are exposed to sunlight such as head, neck, arms, trunk, and legs. Nowadays, manual visual diagnosis of melanomas by trained professionals is most commonly used in the detection and classification of melanomas. Melanoma skin cancer can further be categorized into malignant melanoma and nonmalignant melanoma. Malignant melanoma is the least common but yet the more aggressive of the two types of skin cancer.

Dermoscopy, also called dermatoscopy or skin surface microscopy, is the technique of investigating the skin lesions that helps professionals in cancer examination. Digital dermoscopy is considered to be preferred approach due to its correctness and accuracy in the results. Digital cancer images are used in Computer Aided Diagnostic (CAD) systems for screening of melanoma and its different stages. Different signs of disease appear with different properties on the surface of lesion and it is the goal of CAD systems to identify these signs for timely and accurate treatment of cancer. Automated detection of melanoma is comprised of various steps including preprocessing, extracting region of interest, postprocessing, and finally segmentation.

**Figure 1:**
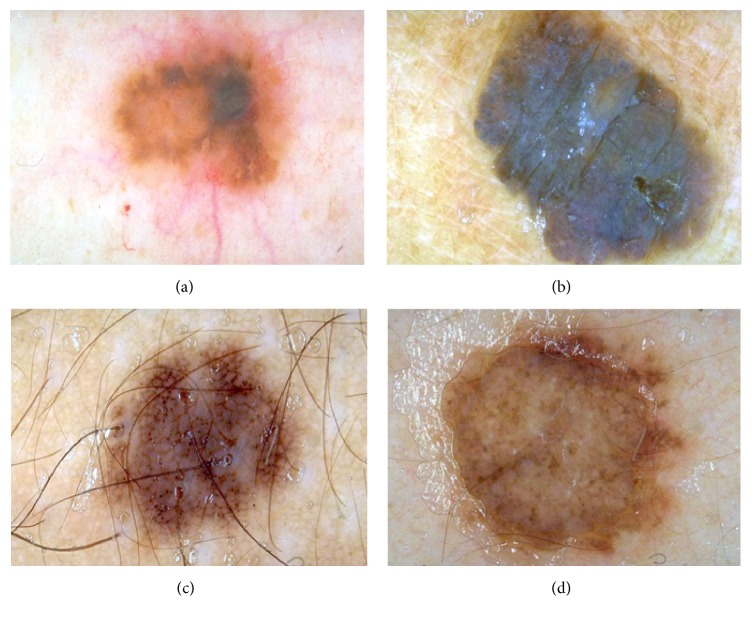
Sample dermoscopic images containing malignant melanoma and highlighting the variations in the melanoma lesions. (a) Smooth transition between lesion and the image. (b) Specular reflection. (c) Presence of hairs. (d) Gel and bubble presence.

Image segmentation is the process of segmenting a digital image into multiple partitions. The aim of segmentation is to filter irrelevant information from the image that is not the part of lesion. Dermoscopic images are generally affected by certain artifacts including smooth transition between lesion and the skin, presence of hairs, transition effects of gel and water bubble, multiple colored lesions, and specular color reflections. Segmentation is further problematic due to irregular shapes [2] and sizes of lesions with different textures and skin types [3, 4]. Some sample digital dermoscopic images of malignant melanoma, highlighting these artifacts, are presented in Figure 1. Preprocessing steps are required to handle these artifacts which will otherwise have negative impact on the feature computation and in turn skin cancer categorization. After removal of problematic artifacts, region of interest from the image is extracted and the lesion is segmented from the image. After segmenting, we try to identify and compute features which makes different categories of melanoma distinct from each other. These features are further used by classifiers such as [5–8] to automatically recognize different types of melanoma. This paper is primarily focused on the first two steps of dermoscopic analysis of melanoma that include preprocessing and segmentation [9]. Image processing approaches [10, 11] and research in related domains such as retinal lesion detection and classification [12–14] are also somewhat relevant to the problem at hand of skin lesion segmentation, feature extraction, and classification.

In this paper, we present our segmentation approach for extracting lesion from skin whilst taking care of the problems of gel, bubbles, hairs, vessels, contrast variations, and other artifacts. The main contribution of the paper is a novel approach for effectively handling the problem of hairs and vessels, enhancing the luminance information, and stretching the contrast between skin and lesion pixels for effective segmentation of lesion. In this paper, we present an effective approach to handle the problems of unwanted artifacts such as hairs and tiny vessels by employing directional wavelet filters and enhancing/detecting pixels representing these artifacts. The detected hairs and vessel pixels are filtered and a novel inpainting approach is presented to fill the missing pixels using neighborhood information. The problem of uneven luminance is also addressed by estimating nonuniform illumination and performing equalization in luminance information. The problem of contrast stretching between skin and lesion is addressed by proposing adaptive sigmoidal function that computes and utilizes cut-off value suitable for individual images. The enhanced image is then processed to segment lesion from skin using combination of thresholding and morphological operations. The proposed approach is tested on the European database of dermoscopic images.

The remainder of the paper is organized as follows. In Section 2, we present a review of recent lesion segment approaches.  Section 3 presents the overview of our proposed lesion segmentation approach. In Section 4, we present our novel methodology for effective detection of hairs and later removing them using proposed inpainting approach.  Section 5 presents our proposed contrast enhancement/stretching and lesion segmentation approach using enhanced dermoscopic image. Experiments are conducted to demonstrate the superiority of the proposed approach as compared with the competitors. These experiments are discussed in Section 6. The last section summarizes the proposed approach and the experimental findings.

## 2. Related Work

To deal with preprocessing and segmentation problems, many algorithms have been proposed that can be broadly classified as thresholding, edge-based, and region-based methods. In [15], a thresholding based segmentation approach is presented. They used combination of global thresholding, adaptive thresholding, and clustering techniques. Their approach attains good results when there is good contrast between the lesion and the skin. However, their approach will not provide impressive performance where there is a poor contrast and smooth transition between skin and lesion which is a norm. In assumption of bimodal distribution, if a global threshold is applied, it will result in poor segmentation results. Abbas et al. [16] presented an automatic segmentation technique based on double thresholding for segmentation.

Edge-based lesion segmentation approaches are presented in [17–19]. Gonzalez and Woods [17] presented an approach for lesion segmentation that is based on the zero-crossings of the Laplacian-of-Gaussian. A variety of active-contour based approaches have been proposed [18, 19]. Argenziano et al. [18] presented gradient vector flow (GVF) based active-contour model whereas Celebi et al. [19] presented geodesic active-contour model and the geodesic edge tracing for lesion segmentation. Edge-based approaches perform poorly when the boundaries of the lesions are not well defined and the color transition between skin and lesion is smooth. Another difficulty is the presence of fake edge points caused by the presence of artifacts such as hair, color reflections, or irregularities in the skin texture which are not the part of the lesion boundary [20].

Region-based approaches have also been employed which include multiscale region growing [21], direction sensitive modified fuzzy* c*-means algorithm [22], morphological flooding [15], multiresolution Markov random field algorithm [18], and statistical region merging [23]. Region-based approaches have difficulties in the presence of variation in color and/or the presence of texture in lesion or skin region leading to oversegmentation. Gómez et al. [24] provide a comparison of different techniques presented to segment lesions in dermoscopic images including adaptive thresholding [25], fuzzy* c*-means [26], spherical coordinate transform (SCT)/center split [27], principal components transform (PCT)/median cut [27], split and merge [28], and multiresolution segmentation [28]. They have not included any edge-based techniques in their comparative analysis.

In the past decades, a variety of techniques have been proposed for skin lesion detection or segmentation [18, 19, 29, 30] based on thresholding, clustering, and region growing in gray scale. Unsupervised techniques based on statistical region merging (SRM) and color-texture (JSEG) algorithms are proposed in [23] and [31], respectively. Abbas et al. [16] proposed a segmentation technique based on region-based active contour (RAC). Lissner and Urban [23] introduced an improved dermatologist-like tumor area extraction (DTAE) algorithm. In addition, the remaining techniques are mostly based on nonuniform color spaces [32, 33].

According to Emre Celebi et al. in [34], in automated skin lesion diagnostic system, boundary detection system and image acquisition process should be described in sufficient detail. Testing should be done from random collection of images from a large and diverse image database. Test set of images should be large enough to ensure statistically valid conclusions. Test image group should not be used to train the boundary detection method. Distribution of the diagnostic image sets should be specified. The algorithm with reasonable computational requirements should be used. The evaluation of the results to determine lesion boundaries should be compared with marked boundaries taken from different dermatologists. The results should be compared with other studies published. The implementation of boundary detection method should be public to improve accessibility and reuse.

Another skin cancer segmentation approach is variational model for image segmentation presented in [35]. Mean shift based gradient vector flow algorithm was validated against competing methods including classical GVF and level set and it provided best accuracy and robustness. Among the most advanced technologies, this method is quite accurate, because it gets iterative energy minimization process the best solution. The algorithm incorporates a function of the mass density with classic GVF term. Achieving this final solution is based on the integration of support functions and mean drift estimate numerical optimization program [27].

## 3. Overview of Proposed Segmentation Framework

**Figure 2:**
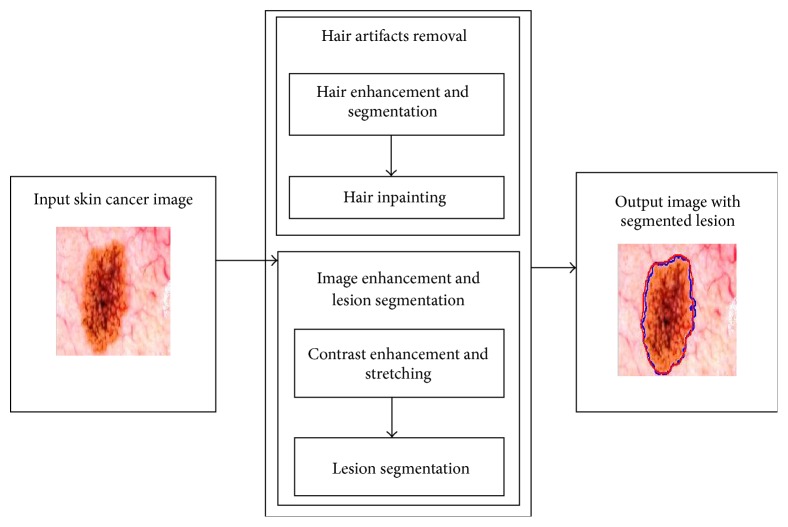
Phases of automated skin lesion segmentation system.

In this section, we present an overview of our proposed approach for the segmentation of melanocytic and nevus lesions from skin whilst handling the problems of hairs, gel, inconsistent contrast, and other artifacts. The proposed algorithm is composed of three major steps including hair detection and inpainting, color space transformation, and contrast stretching/enhancement and then finally segmentation of the lesion area. The flow diagram of the proposed methodology is presented in Figure 2. The proposed system takes a dermoscopic image as an input and removes major unwanted artifact of hairs to avoid its effect on subsequent steps of image enhancement and segmentation. Hair removal is achieved by highlighting and detecting hairs and further removing them by inpainting the hair pixels using their neighborhood information. The system then performs desired color space transformation and performs image enhancement on the selected component of color space to better differentiate between skin and lesion. Lesions are then segmented from the background skin image in the final step.

## 4. Hair Artifacts Removal

**Figure 3:**
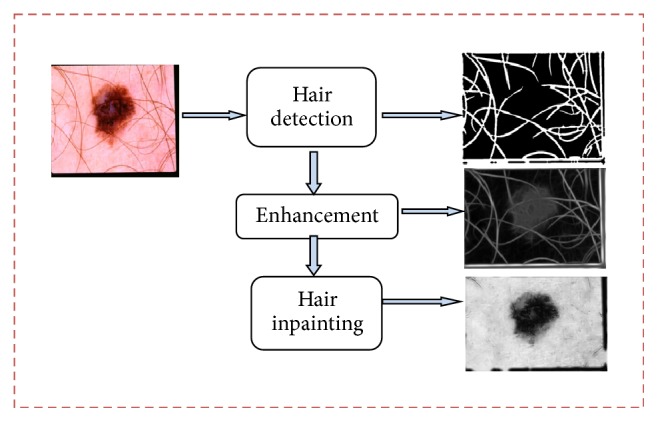
Flow diagram for hair artifacts removal algorithm.

In this section, we present our proposed approach for removal of hair artifacts before segmentation of lesions. This is a classification-free method which enhances the hair information using Gabor wavelet-based directional filters/enhancement and then mitigates its effect using inpainting based segmentation technique employing neighborhood estimation. The proposed approach for hair artifact removal focuses on reducing the effect of hair in lesion segmentation which appears as false positive and degrades system performance.  Figure 3 shows flow diagram of proposed system for hair artifact removal. Hair artifact removal method first detects all the hairs present in image and creates a binary mask of all hairs. The detected hair regions are then filled using an algorithm of Neighborhood Based Region Filling (NBRF). The key idea of this method is to inpaint the hairs before lesion enhancement and segmentation so as to avoid the enhancement of artifacts upon applying enhancement and segmentation techniques.

### 4.1. Hair Enhancement and Segmentation

The major artifact that creates problem in lesion segmentation is the presence of thin hairs in the image. So it is necessary to improve the image pattern. We have used 2D Gabor wavelet to enhance the image pattern and highlight the hair definition [25]. These wavelets are the best option to tune in frequencies due to their potential of fine feature detection. In order to apply Gabor wavelet, we used continuous time wavelet transformation (CWT) [26]. The 2D CWT *T*_Φ_(**b**, *θ*, *a*) is defined in terms of the scalar product of *f*(*x*, *y*) with the transformed wavelet Φ_**b**,*θ*,*a*_(**x**):

(1) TΦb,θ,a=WΦ−1/2a∫exp⁡jRbΦ^∗ar−θRf^Rd2R.

Here, $j=\sqrt{-1}$ and $\hat{\Phi }$ is Fourier transform of Φ. *W*_Φ_, Φ^*∗*^, **b**, *θ*, and *a* denote the normalizing constant, complex conjugate of Φ, the displacement vector, the rotation angle, and the dilation parameter, respectively. *r*_−*θ*_ is two-dimensional rotation along** x**. The mathematical functions for Gabor wavelet and its Fourier transform are defined as

(2) ΦGaborx=exp⁡jR0xexp⁡−12Ax2,Φ^Gaborx=det A−11/2exp⁡−12A−1R−R02,

where **x** = [*x* *y*]^*T*^ and **R**_0_ is a vector that defines the frequency of the complex exponential. $A=\left[\begin{array}{cc} \epsilon^{-1/2} & 0\\ 0 & 1 \end{array}\right]$ with elongation *ϵ* ≥ 1 is a 2 × 2 positive definite diagonal matrix which defines the wavelet anisotropy and elongation of filter in any desired direction. For each pixel position with fixed values of **b** and *a*, the Gabor wavelet transform *T*_Φ_(**b**, *θ*, *a*) is computed for *θ* spanning from 0° up to 165° at steps of 15° and the maximum is taken:

(3) $$\begin{array}{c} M_{\Phi }\left(\;\theta \;\right)=\max\left|\;T_{\Phi }\left(\;b,\theta ,a\;\right)\;\right|. \end{array}$$

Table 1 shows the values for Gabor wavelet parameters which are used for enhancement of hairs.

**Figure 4:**
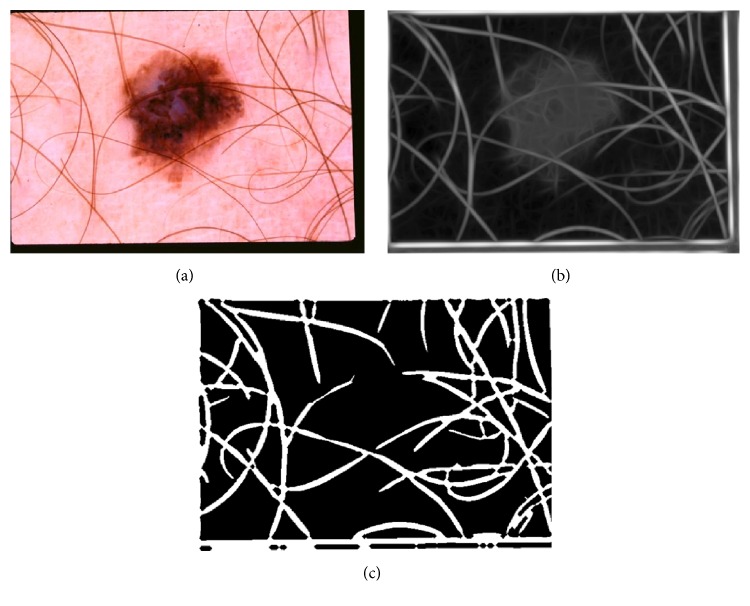
Hair detection. (a) Original image. (b) Hair enhancement using Gabor wavelet. (c) Binary hair mask using adaptive thresholding.

The next step is to make binary mask of hair and it is done by applying adaptive OTSU's thresholding algorithm [14].  Figure 4 shows hair enhancement and binary mask generation results.

### 4.2. Hair Inpainting

**Figure 5:**
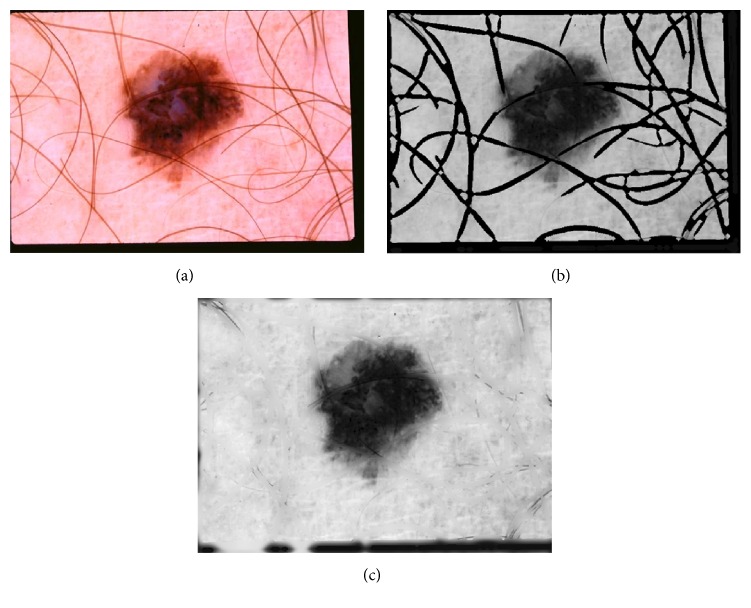
(a) Original image. (b) Image after removal of hair pixels. (c) Image after filling hair pixels using NBRF.

Hair detection module makes a binary mask for all hairs present in the image. The next step is to remove all hair pixels and fill those pixels to have a smooth image. Hair inpainting step takes binary hair mask as an input and fills all the hair regions in a very smooth manner by using an algorithm of Neighborhood Based Region Filling (NBRF). NBRF algorithm works in radial way towards the center of the objects. It fills hairs in a very smooth way by estimating the neighborhood and averaging the background. It runs iteratively which eventually blends all hairs within the filled region.  Algorithm 1 shows the algorithm for NBRF. The algorithm runs in a recursive manner and fills all hair pixels using morphological operations until all pixels are filled.  Figure 5 shows the results of image with inpainted hairs obtained after applying NBRF algorithm.

## 5. Lesion Segmentation after Contrast Enhancement and Stretching in Luminance (*L*) Space

In this section, we present our approach for image enhancement by performing contrast enhancement and stretching. Our proposed approach operates on the luminance (*L*) component of the *L* ^*∗*^ *a* ^*∗*^ *b* ^*∗*^ color space. It has been observed that there is a wide variation in luminance information of different images. Similarly, the contrast between the skin and the lesion is critically dependent on lighting conditions and tone of skin and lesion pixels. This results in significant variation in contrasts of skin and lesion pixels over different dermoscopic images. Equalization of luminance and enhancement of contrast between skin and lesion pixel are therefore critical for the success of lesion segmentation algorithm.

### 5.1. Contrast Enhancement

In this section, we present an approach for correction of illumination in the extracted* L* channel of the image based on imaging condition and the geometry knowledge to provide a visually standard value of lightning for all of the images. This approach can be trivially extended to correct the color components of the dermoscopic images if required. The proposed solution works with the following steps.

#### 5.1.1. Estimation of Nonuniform Illumination in* L* Channel

This phase takes luminance (*L*) channel as input and performs the nonuniform illumination estimation present in* L* channel of the image using the following steps:

(1) Compute initial background mask (potentially representing the skin pixels) as follows:
  (4) $$\begin{array}{c} bg\left(\;x,y\;\right)=\left\{\begin{array}{cc} 1 & iff\;\;L\left(x,y\right)>1.2*thresh\left(L\right)\\ 0 & otherwise, \end{array}\right. \end{array}$$
  where thresh(·) is a function that automatically detects a threshold value to binarize gray scale image as proposed in [36].
(2) Calculate *u*_*L*_(*x*, *y*) as follows:
  (5) $$\begin{array}{c} u_{L}\left(\;x,y\;\right)=\frac{\left(L\left(x,y\right)*bg\left(x,y\right)\right)⊕G_{m}\left(x,y\right)}{bg\left(x,y\right)⊕G_{m}\left(x,y\right)}, \end{array}$$
  where
  (6) Gmn1,n2=−n12+n22−2σ2hgn1,n22πσ6∑n1∑n2hgn1,n2,hgn1,n2=exp⁡−n12+n222σ2
  is Gaussian kernel, with the standardized parameters.
(3) Compute the illumination estimation *E*_*L*_ as follows:
  (7) $$\begin{array}{c} E_{L}\left(\;x,y\;\right)=\frac{G_{fm}G\left(x,y\right)*u\left(x,y\right)⊕G_{m}\left(x,y\right)}{G_{fm}\left(x,y\right)⊕G_{m}\left(x,y\right)}, \end{array}$$
  where
  (8) $$\begin{array}{c} G_{fm}\left(\;x,y\;\right)=\left(\;u_{L}\left(\;x,y\;\right)-L\left(\;x,y\;\right)\leq 0.05\;\right)*bg\left(\;x,y\;\right). \end{array}$$

#### 5.1.2. Equalization of* L* Channel

The estimated nonuniform luminance distribution component *E*_*L*_, computed using (7), is then used to generate the equalized* L* channel (*L*_eq_) as follows:

(9) $$\begin{array}{c} L_{eq}\left(\;x,y\;\right)=L_{ref}*\frac{L\left(x,y\right)}{E_{L}\left(x,y\right)}, \end{array}$$

where *L*_ref_ is a constant used to adjust the overall illumination of the image. We assume *L*_ref_ = 0.59 based on empirical evaluation.

#### 5.1.3. Contrast Stretching

In this section, we present our proposed approach to stretch the contrast of the dermoscopic image to enhance the illumination variation between skin and lesion pixels. To achieve this, we normalize the equalized luminance image (*L*_eq_) using

(10) Leqx,y=Leqx,y−Leq_minkLeq_max−Leq_minfor  Leq_min≤Leqx,y≤Leq_max,

where *L*_eq_min_ and *L*_eq_max_ are the 1st and 99th percentile values, respectively, within the equalized* L* component *L*_eq_(*x*, *y*) and *k* is the constant that determines the intensity of skin pixels other than lesions. The value of *k* is determined empirically and is set to 0.75.

**Figure 6:**
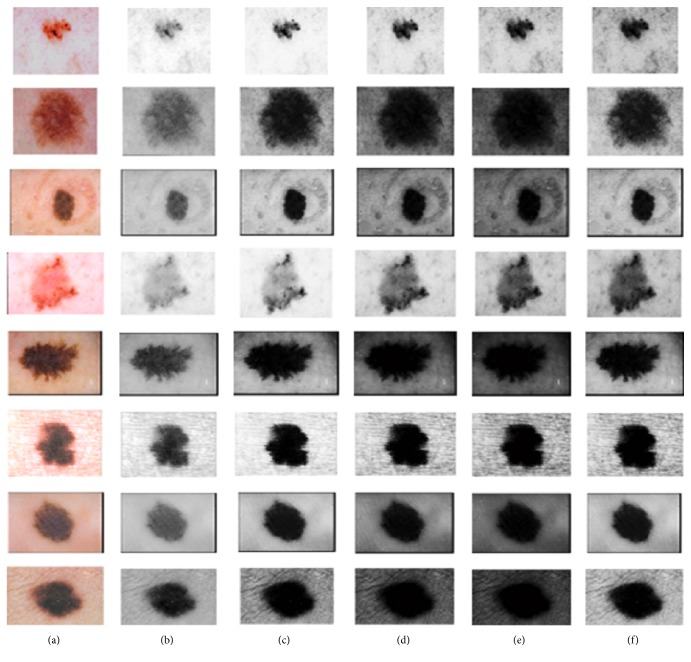
Qualitative analysis of proposed adaptive sigmoidal function. (a) Original images. (b) Before applying sigmoidal function. Contrast stretching achieved after applying sigmoidal function with (c) *δ* = 0.65, (d) *δ* = 0.75, and (e) *δ* = 0.8. (f) Proposed adaptive value for cut-off *δ*.

The contrast between the luminance of skin and lesion pixels is enhanced by employing adaptive sigmoidal using cut-off value computed separately for each dermoscopic image. A good value for cut-off *δ* is approximated by computing cumulative histogram of *L*_eq_ image. We can employ *b*-bit quantization for the computation of cumulative histogram which implies that the histogram of *L*_eq_ component is quantized using 2^*b*^ bins. Employing higher number of bins makes the proposed approach sensitive to local fluctuations in histogram whereas lower number of bins will result in rough approximation of *δ*. We have employed *b* = 4 based on empirical evaluation. Let CH_*L*_ represent the cumulative histogram of enhanced luminance component of the image (*L*_eq_); the value for *δ* is approximated as follows:

(11) $$\begin{array}{c} \delta =℘*\underset{\left(i\right)}{\arg\;\max}\left(\;{CH}_{L}^{\prime }\left(\;i\;\right)\;\right)*2^{b}, \end{array}$$

where *i* is the bin index of histogram CH_*L*_, CH_*L*_′ is first-order derivative of CH_*L*_, and *℘* is the scaling parameter with values 0 ≤ *℘* ≤ 1. Lower values of *℘* result in enhancing the luminance of even relatively darker pixels (representing part of lesions) to be detected as part of the skin and vice versa. The value of *℘* is determined empirically and is set to 0.88. We then update the luminance information by applying adaptive sigmoidal function based on the dynamic cut-off value *δ*, as learned using (11), as follows:

(12) $$\begin{array}{c} L\left(\;x,y\;\right)=\frac{1}{1+\exp\left(gain*\left(\delta -L\left(x,y\right)\right)\right)}\;\forall x,y, \end{array}$$

where the value of gain is determined empirically and we set gain = 10. Qualitative analysis of adaptive sigmoidal function as compared to traditional sigmoidal function with varying cut-off values is presented in Figure 6. It is apparent from Figure 6 that the static value for cut-off gives good contrast stretching between lesion and skin pixels for some images but deteriorates the contrast in other images. On the other hand, computation of dynamic cut-off in adaptive sigmoidal function enhances the contrast for all the dermoscopic images.

### 5.2. Lesion Segmentation

After contrast enhancement and stretching of luminance component, the target lesion differs greatly in contrast from the skin part. We further apply median filtering (*L*_*M*_ = median_filtering  (*L*_eq_)) with a disk shaped structuring element to handle the presence of fine-level noise. We then perform a sequence of morphological operations to make the lesion definition more clear to be robustly segmented from the skin. We first employ morphological erosion operation on *L* channel (*L*_*O*_ = open  (*L*_*M*_)) using a disk shaped structuring element. *L*_*O*_ is later used as a marker to perform morphological reconstruction with the median filtered luminance image (*L*_*M*_) as the mask. Morphological reconstruction can be thought of conceptually as repeated dilations of marker image, until the contour of the marker image fits under mask image. Morphological reconstruction has been employed using 8-connective neighborhood (*L*_MC_ = morphological_reconstruction  (*L*_*M*_)). In morphological reconstruction, the peaks in the marker image spread out or dilate. This process is followed by closing and erosion by a relatively smaller disk shaped structuring element to merge pixels at the border of lesion whilst eliminating fine-level noise elsewhere. This will have a net effect of removing small blemishes without affecting the overall shapes of the lesion object. The processed image is then binarized as follows:

(13) LBx,y=1LMCx,y<threshLB−κ0otherwise∀x,y,

where thresh(·) is a function that automatically detects a threshold value to binarize based on the histogram of *L*_MC_ as proposed in [36] and *κ* is a constant which determines the sensitivity of our proposed segmentation algorithm to detect the boundary pixels of lesions as foreground or background. As there is sometimes a smooth transition from lesion pixels to skin pixels, lower values of *κ* will result in shrinking of lesion regions and result in boundary pixels to be classified as skin region and vice versa. We assumed *κ* = 0.08 based on empirical evaluation.

We than employ connected component labelling using 8-connective neighborhood to identify distinct object in *L*_*B*_. Let **O** be the list of detected objects (inclusive of noisy objects); the filtered list by removing the unwanted objects **O**_filtered_ is then generated as follows:

(14) Ofiltered=Ofiltered∈O ∣ ∀O∈Ofiltered,  O<20∧SolidityOi>0.25,

where |·| is a pixel count function for a given object and Solidity(·) is a function that computes the proportion of the pixels in the convex hull that are also in the object. The pixels belonging to object/objects in **O**_filtered_ are the segmented pixels representing the lesion in the image.

## 6. Results and Discussion

In this section, we present results to demonstrate the effectiveness of proposed approach for the segmentation of melanocytic and nevus lesions in the presence of unwanted artifacts of hairs, gel, illumination variation, and so forth. Various experiments are performed to evaluate the validity of the proposed approach and to provide comparison with existing approaches. The lesion segmentation technique is tested effectively on dataset of a total of 100 dermoscopy images. This dataset consists of both invasive malignant melanoma and benign lesions.

### 6.1. Performance Metrics

The performance metrics used for quantitative analysis of proposed approach and its comparison with the competitors are True Positive Rate (TPR), False Positive Rate (FPR), and error probability (EP) measures. These evaluations metrics are computed as follows:

(15) True  Detection  RateTDR=TPTP+FN∗100,False  Positive  RateFPR=TNFP+TN∗100,Error  rateER=FP+FNTP+TN+FP+FN∗100,

where, TP, TN, FP, and FN are true positives, true negatives, false positive, and false negatives, respectively.

**Experiment 1 (evaluation of proposed hair artifact removal and image inpainting approach).**

**Figure 7:**
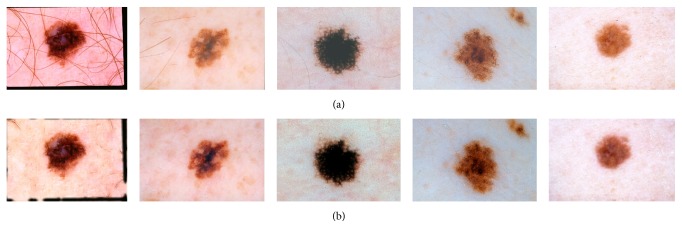
Qualitative evaluation of proposed hair artifact detection and image inpainting approach. (a) Original image with hair artifacts. (b) Corresponding processed images after hair removal.

The purpose of this experiment is to perform a qualitative evaluation of our proposed approach to remove the hair artifacts from the dermoscopic images to mitigate its effect on lesion segmentation. The proposed approach whilst filtering the noisy hair pixels fills filtered pixels using the neighboring skin pixels.  Figure 7 highlights the effectiveness of the proposed hair detection and inpainting approach, as presented in Section 4 on various dermoscopic images containing hair artifacts. The images containing varying density of hairs are selected to highlight the performance of proposed approach on different possible scenarios of the presence of hair artifacts. As obvious from Figure 7, the proposed approach successfully detects all hairs in the image and then correctly replaces them with the skin information from the neighboring skin pixels whilst having minimal side effects of hair removal. The successful removal of hair artifacts contributes significantly to the segmentation of lesions from skin.

**Experiment 2 (quantitative evaluation of proposed contrast enhancement and adaptive contrast stretching).**

The purpose of this experiment is to analyze the contribution of proposed contrast enhancement and stretching approach in the effectiveness of lesion segmentation. To perform the analysis of contrast enhancement component as proposed in Section 5.1, we performed complete lesion segmentation process, compared the segmentation results with ground truth, and computed the performance metrics as discussed in Section 6.1. The experiments are repeated with different static values of cut-off and other parameters employed in sigmoidal function, typically used in literature, and are compared with the dynamic values of these parameters computed as proposed in Section 5.1. Performance metrics of TDR, FPR, and ER, computed for these different settings, are presented in Table 2. Ideally, we want higher value of TDR (True Detection Rate) and lower values of FPR (False Positive Rate) and ER (error rate). As obvious from the results, the proposed dynamic computation of cut-off parameter in sigmoidal function significantly enhances the performance of proposed system as compared to user-specified global cut-off values for all images. This improved performance is explained by the fact that each image has different luminance and significant contrast variations between skin and lesion. Thus, a static cut-off value working good for one image may give poor results for the other images. These phenomena have been highlighted in Figure 6 through qualitative results. The proposed approach to employ dynamically computed value of cut-off results in overall good values for TDR, FPR, and EP. Setting cut-off value to 0.8 resulted in the highest TDR but results in lots of false positives and hence poor FPR and EP values. On the other hand, cut-off value of 0.65 resulted in lowering of FPR and EP values but significantly degrading the TDR.

**Experiment 3 (comparison of proposed approach with competitors).**

**Figure 8:**
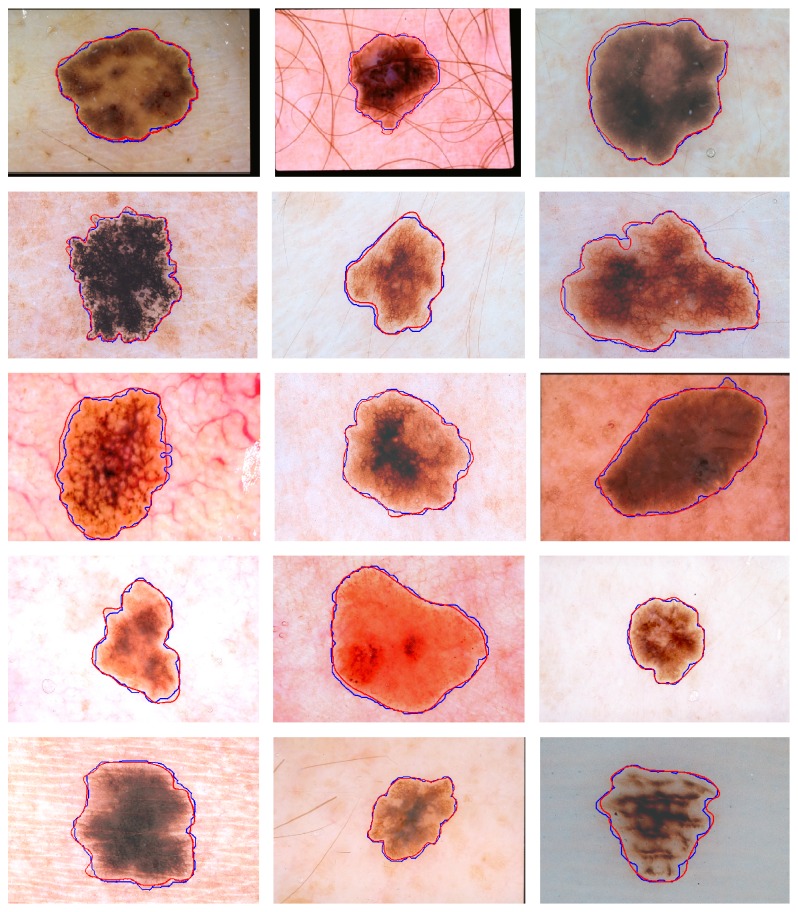
Lesion segmentation using proposed approach (blue contour) along with ground truth (red contour) imposed on various dermoscopic images.

In this experiment, we compare the performance of proposed lesion segmentation approach with existing approaches including melanoma border detection (MBD) algorithm, color-texture algorithm (JSeg), dermatologist tumor area (DTEA) detection, and region-based active-contour (RAC) algorithm [16, 18, 19, 30]. The lesion segmentation is carried out using 100 dermoscopic images and the performance metrics of TDR, FPR, and EP, computed for various algorithms, are presented in Table 3. It is observed that the proposed approach achieves the higher average value of TDR of 97.26%, value of FPR of 3.52%, and error probability of 3.01%. The obtained results indicate that the proposed algorithm obtains better melanoma border detection results, which are significant, compared with other three state-of-the-art techniques. Better results of the proposed approach can be attributed to the removal of hair artifacts and dynamic nature of contrast enhancement and stretching which results in significant variation in skin and lesion pixels to be identified with significant degree of accuracy.  Figure 8 shows some qualitative results to demonstrate the effectiveness of the proposed lesion segmentation approach. The red outline of the lesion depicts the manual segmentation done by dermatologist and blue outline represents the segmentation achieved by the proposed system. It is clear from the figure that the proposed approach gives good segmentation results whilst handling the artifacts of hairs, veins, gel, and varying luminance and contrasts. However, still there are minor over- and undersegmentation of lesion. These are normally caused by variation in color and intensity within the lesion that matches with the background skin. Adaptive sigmoidal function occasionally suppresses the pixels of multitone lesions. These pixels match the skin or some artifacts in the skin which might not be removed by preprocessing and falsely appear as part of lesion. Overall, the proposed approach gives good performance as depicted by results presented in Figure 8.

**Figure 9:**
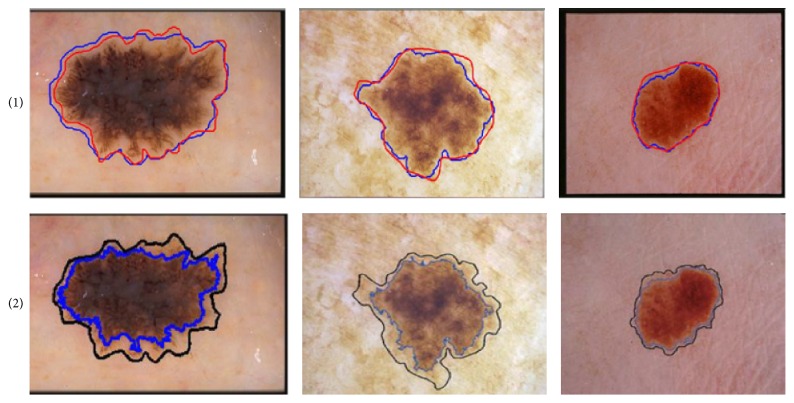
Segmentation comparison results. (a) Results of our proposed method. (b) Results of MBD technique.

Figure 9 shows the pictorial comparison of proposed system with MBD as compared to the ground truth. For the competitor's result, the black outline represents ground truth and the blue outline represents the segmentation results obtained using MBD. The difference between ground truth and MBD technique is clearly highlighted in the figure. The proposed method gives almost the same boundary as given in the ground truth.

In order to compute the computational time of proposed system, we run our algorithm and existing algorithms on Core i-5 2.1 Ghz system with 4 GB RAM. The average processing time of the proposed approach is 1.76 sec. Many other techniques are doing their jobs well but complexity of the computation has become their drawback. The processing time of MBD is 10 sec. Similarly, other techniques' processing time is as follows: classical GVF: 12 sec, level sets: 14 sec, MGVF: 17 sec, MSGVF: 22 sec, and so forth. Our proposed technique is efficient enough with respect to computation time.

## 7. Conclusion

In this paper, we have proposed a novel method of segmenting melanocytic and nevus lesions from skin using dermoscopic images. The proposed approach handles the problems of unwanted artifacts such as hairs and tiny vessels by employing directional wavelet filters, enhancing and detecting pixels representing these artifacts. The detected and filtered hairs and vessel pixels and the missing pixels are inpainted using neighborhood information using our proposed NBRF algorithm. The problem of uneven luminance is also addressed by estimating nonuniform illumination and performing equalization in luminance information. The contrast stretching between skin and lesion is further done using proposed adaptive sigmoidal function that computes and utilizes cut-off value suitable for individual images. The enhanced image is then processed to segment lesion from skin using combination of thresholding and morphological operations. The proposed algorithm was tested on 100 dermoscopy images containing invasive malignant melanoma, nevus, and benign lesions. The performance of the proposed approach is compared with existing approaches (JSeg, DTEA, and RAC). The experimental results, as presented in Section 7, demonstrate the superiority of the proposed approach as compared with competitors. The proposed approach gives the best values of performance metrics with TDR of 97.2618%, FPR of 3.62%, and error probability of 3.39%. Qualitative results of segmentation as presented in Figure 8 depict the ability of proposed approach to handle the problems of hairs and vessels and problems associated with luminance and contrasts. Figure 9 further highlights the superiority of proposed approach as compared to the closest competitor (MBD). The experimental results give evidence to the claim that the proposed lesion segmentation approach is very effective approach that is robust to the presence of unwanted artifacts of hairs, vessels, variable luminance, and contrasts.

## Figures and Tables

**Algorithm 1:**
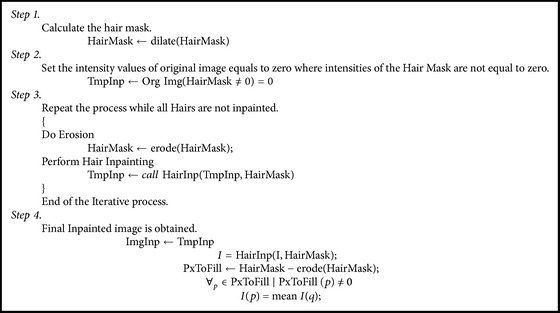
Stepwise specification of NBRF Algorithm.

**Table 1:** Parameter values for Gabor wavelet.

Parameter	Value
Dilation (*a*)	2.5
Elongation (*ϵ*)	3
Rotation angle (*θ*)	15°
*k*_0_	[0,3]

**Table 2:** Performance analysis of proposed dynamic contrast enhancement approach as compared to static alternative.

Cut-off value	TDR%	FPR%	EP%
0.55	88.08	2.26	4.91
0.65	91.56	3.0	4.52
0.71	95.95	4.54	4.52
0.75	97.28	5.20	4.67
0.80	97.33	6.10	5.47
Adaptive value	97.26	3.52	3.01

**Table 3:** Performance comparison of proposed technique with other methods of segmentation.

Cut-off value	TDR%	FPR%	EP%
JSeg	84.20	12.46	15
DTAE	87.50	9.78	13
RAC	89.41	8.34	07
MBD	94.25	3.56	04
Proposed method with cut-off = 0.75	97.28	5.20	4.67
Proposed method with adaptive cut-off	97.26	3.52	3.01
